# Dynamic K-edge Subtraction Fluoroscopy at a Compact Inverse-Compton Synchrotron X-ray Source

**DOI:** 10.1038/s41598-020-66414-x

**Published:** 2020-06-15

**Authors:** Stephanie Kulpe, Martin Dierolf, Benedikt Günther, Johannes Brantl, Madleen Busse, Klaus Achterhold, Bernhard Gleich, Franz Pfeiffer, Daniela Pfeiffer

**Affiliations:** 1 Chair of Biomedical Physics, Department of Physics, Technical University of Munich, James-Franck-Str. 1, 85748 Garching, Germany; 2 Munich School of BioEngineering, Technical University of Munich, Boltzmannstr. 11, 85748 Garching, Germany; 3 Department of Diagnostic and Interventional Radiology, Munich School of Medicine and Klinikum rechts der Isar, Ismaniger Str. 22, 81675 Munich, Germany

**Keywords:** Applied physics, Gastroenterology, Imaging techniques

## Abstract

X-ray fluoroscopy is a commonly applied diagnostic tool for morphological and functional evaluation of the intestine in clinical routine. Acquisition of repetitive X-ray images following oral or rectal application of iodine contrast agent visualizes the time dependent distribution of the contrast medium, and helps to detect for example leakages, tumors or functional disorders. However, movements of the intestine and air trapped inside usually prevent temporal subtraction imaging to be applied to fluoroscopy of the gastrointestinal tract. K-edge subtraction (KES) imaging would enable subtraction fluoroscopy because it allows for imaging of moving organs with little artefacts. Although KES imaging is a well established technique at synchrotron sources, this imaging method is not applied in clinical routine as it relies on brilliant synchrotron radiation. Recently emerging compact synchrotron X-ray sources could provide a quasi-monochromatic, high-flux X-ray beam and allow for the application of KES in a laboratory environment. Here, we present a filter-based dynamic KES approach at the Munich Compact Light Source (MuCLS), the first user-dedicated installation of a compact synchrotron X-ray source worldwide. Compared to conventional temporal subtraction X-ray radiography, our approach increases the contrast while reducing the generated image artefacts.

## Introduction

Contrast enhanced fluoroscopy is an X-ray based imaging technique which is commonly used for visualization of the gastrointestinal tract. Repetitive X-ray images are acquired before and after application of oral or rectal contrast medium, and the diagnosis is made by evaluation of the spatial and temporal distribution of the contrast material. Upper gastrointestinal X-ray series illustrate the way of the contrast medium from the mouth down to the stomach, and movement of the larynx as well as the oesophagus are an important physiological finding during the process of swallowing and therefore, are of special interest in clinical fluoroscopy. As fluoroscopy is a study of moving body structures with a focus on physiological or pathological movements of the organs of the gastrointestinal system, subtraction imaging techniques cannot be used in clinical fluoroscopy of the gastrointestinal tract because of these physiological movements during the imaging procedure. In contrast, digital subtraction angiography (DSA) is a clinically well-established fluoroscopy technique in interventional angiography, which allows for improved visualization of the blood vessels by using a temporal subtraction technique^1^. In this procedure, a mask image without contrast agent is subtracted from all subsequent contrast enhanced images in order to remove background structures and therefore improve the visibility of contrast enhanced vessels. However, artefacts from patient movement, breathing and cardiac motion may still have a negative impact on image quality^2,3^. Therefore, when imaging the gastrointestinal tract, conventional temporal subtraction cannot be used because the intestinal peristalsis, in addition to the movement of air in the intestine and the contractions of the oesophagus, stomach, small bowel and colon will lead to artefacts that impair image quality and diagnostic accuracy^4^.

To improve the image quality in subtraction imaging, K-edge subtraction (KES) imaging, first proposed by B. Jacobson in 1953^5^, has been developed at synchrotron sources. In clinical temporal digital subtraction the images are acquired at the same X-ray energy using a polychromatic X-ray spectrum at different points in time before and after injection of a contrast agent. In contrast, KES imaging exploits the sharp increase of the photoelectric absorption at the K-edge of the contrast agent to enhance the contrast between contrasted structures and surrounding tissue thereby acquiring images directly one after another or, with special setups, simultaneously. By logarithmically subtracting two images taken at X-ray energies bracketing the K-edge of the contrast agent, an image can be calculated which contains only the contrasted structures without background signal because the absorption signals of uncontrasted surrounding tissues barely differ in both images and cancel out in the subtraction^5,6^. While K-edge subtraction works best using a monochromatic X-ray beam, it has also been performed at conventional polychromatic laboratory sources using a Ross filter arrangement^7^ or a multi-bin photon counting detector^8,9^. While the use of filter pairs provides a well-defined and sharp energy window^7^, the absorption of a large amount of the X-ray flux in the filters leads to long acquisition times limiting the applicability for fast X-ray imaging. Detectors with multiple energy bins can also be used to acquire images around the K-edge of a material. However, these detectors usually have an energy threshold resolution of 1–2 keV^10,11^, which limits the ability to acquire images closely around the K-edge. KES imaging can also be performed at conventional polychromatic X-ray sources using 2D pixelated spectroscopic detectors^12,13^ with the disadvantage that in some applications the sample is irradiated with a much broader spectrum than needed for the selected energy bins causing unnecessary radiation dose. In the past, KES imaging has mainly been performed at synchrotrons, where it has been widely applied both in radiography^14–17^ and computed tomography^18–22^. KES imaging at synchrotrons provides better image quality in comparison to temporal subtraction at conventional polychromatic sources after intravenous injection of contrast agent^23^. Additionally, Elleaume *et al*. showed that KES is more suitable for moving organs than conventional temporal subtraction^24^. KES with a gaseous contrast agent like Xe provides similar advantages of high spatial and temporal resolution in functional lung imaging (mostly of small animal models so far) over the conventional techniques (MRI, SPECT, PET) employed therein^22^. Additionally, KES fluoroscopy can be performed with minimal artefacts^25^ and therefore enable subtraction imaging for applications for which conventional temporal subtraction is not useful.

Notwithstanding, the dependence of KES imaging on monochromatic X-rays provided at large scale synchrotron facilities and the limited access to these facilities inhibit widespread application of this technique in clinical routine. This issue may be overcome with compact synchrotron X-ray sources, which are actively developed by multiple groups worldwide with the goal to provide brilliant X-rays in a laboratory frame^26–28^. One of these sources is installed at the Munich Compact Light Source (MuCLS). Here, a Compact Light Source (CLS, Lyncean Technologies Inc., Fremont, USA) produces quasi-monochromatic X-rays with tunable energy through the process of inverse Compton scattering^29^. In past experiments, it has been shown that monochromatic angiography at the MuCLS produces images with improved contrast-to-noise ratio (CNR) compared to images produced with a conventional polychromatic X-ray spectrum^30^. When expanding the procedure to KES angiography, it has been shown that a filter-based KES method at this laboratory source improves the visibility of small blood vessels that are overlaid by bone structures^31^ and allows for the separation of calcium in kidney stones and iodine in computed tomography^32^. To this end, the X-ray energy of the source is not changed directly but by inserting an X-ray filter containing the same material as the used contrast agent. This allows for a fast change in energy since the filter mostly absorbs the part of the spectrum above the K-edge of the contrast agent and shifts the mean energy of the remaining spectrum to lower energies.

Here, we present a filter-based KES fluoroscopy application and evaluate its performance compared to conventional temporal subtraction at the MuCLS. For this, iodine contrast agent was injected into an *ex vivo* mouse while acquiring X-ray projection images that were subsequently filtered and not filtered with an iodine filter. From the acquired images, KES and conventional temporal subtraction images were calculated. At the same time, movement of the sample was simulated to demonstrate the appearance of motion artefacts commonly observed in *in vivo* conventional temporal subtraction imaging. The results imply that KES allows for imaging with reduced or no artefacts which would enable subtraction imaging of e.g. the gastrointestinal tract in future medical applications.

## Results

**Figure 1:**
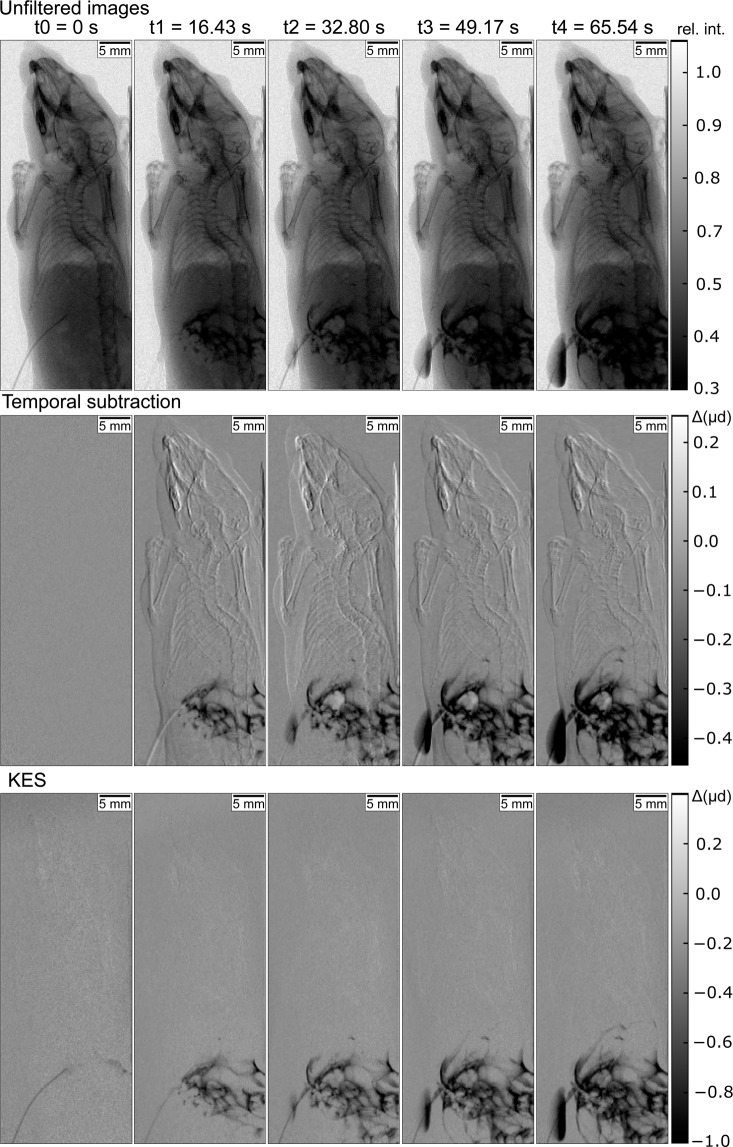
Unfiltered, conventional temporal subtraction and K-edge subtraction (KES) images of an *ex vivo* mouse, in which iodine contrast agent has been injected at time point *t*_0_ in the abdominal peritoneal region. In the conventional fluoroscopic X-ray image the contrast between contrast agent and surrounding bone and tissue is compromised. Both KES and conventional temporal subtraction are able to remove surrounding tissue and bone structures, yet conventional temporal subtraction is prone to artefacts, when there is sample movement between the images that are being subtracted. In this case the movement was simulated by shifting the images with a sinusoidal function continuously to the left and right. The gray scales for the unfiltered images display the relative intensity/transmission of the X-ray beam, while the gray values in the KES and conventional temporal subtraction images show the negative differences in the absorption Δ(*μd*) with *μ* being the mean absorption coefficient of the sample in one pixel and *d* being the thickness of the sample.

The unfiltered images are shown in Fig. 1 together with the calculated conventional temporal subtraction and KES images at different points in time (for full time range see Supplementary Videos S1, S2 and S3 showing the complete non-subtraction, temporal subtraction and K-edge subtraction image series, respectively). The unfiltered images are comparable to clinical fluoroscopy images of the gastrointestinal tract where no subtraction imaging is performed and the contrast between the iodine contrast agent and surrounding bone and tissue structures is limited. In conventional temporal subtraction, only the unfiltered images are needed for the processing, where the first image at *t* = 0 s is logarithmized and subtracted from all following unfiltered images. For each KES image, two subsequently acquired unfiltered and iodine-filtered images were subtracted according to the scheme presented in the Methods section so that all regions that contain contrast agent are highlighted. To simulate the movement of the body through breathing and of the organs inside, movement was simulated by shifting the images with a sinusoidal function continuously to the left and right before further processing of the images, both for KES and conventional temporal subtraction.

Both KES and conventional temporal subtraction are able to enhance the contrast between the iodine contrast agent and uncontrasted structures so that the contrast agent is clearly visible on top of background structures and its temporal dispersion can be observed. Yet, small movements of the specimen can be seen in the conventional temporal subtraction images whereas they do not show up in the KES images. This is a major issue in clinical temporal subtraction imaging and limits the application of this imaging method since artefacts from complex movements of organs such as the intestine and the oesophagus cannot be corrected. In KES, motion artefacts do not occur since the time difference between the two subtracted images is very small (33 ms in this case). Since the time difference between the subtracted images increases in conventional temporal subtraction with every further image, motion artefacts usually increase over time. Only at time points where – in this simple case of a perfectly periodic motion—the sample is very near to the initial position, as at t = t2, there are less artefacts (cf. Fig. 1).

**Figure 2:**
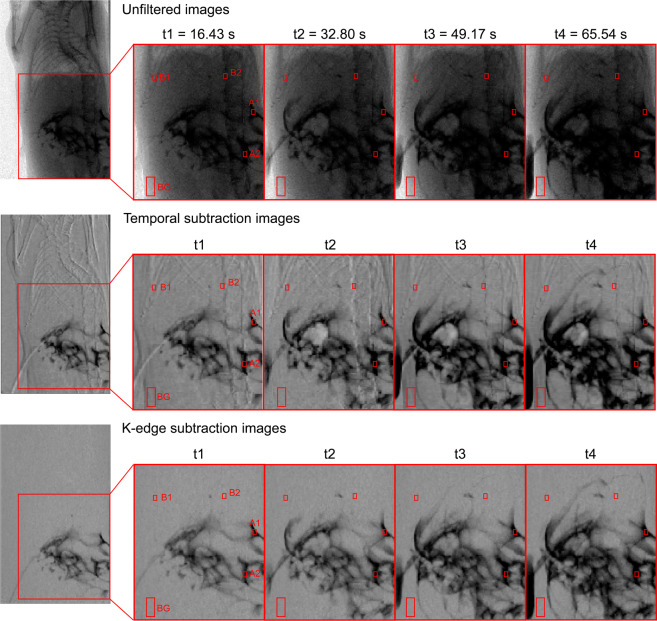
Zoom into regions of Fig. 1 to show regions of interest (in the abdominal peritoneal region) in unfiltered, KES and conventional temporal subtraction images used to calculate the CNR between iodine contrast agent and bone structures. Regions A1 and A2 contain iodine contrast agent, while B1 and B2 are regions with rib and back bone structures, respectively. The contrast was calculated versus the noise in the larger background region BG.

To compare the visibility of the iodine contrast agent, the CNR was calculated between iodine contrast agent and bone structures for two different regions shown in Fig. 2. Thereby, the CNR between regions A1 and B1 (rib structure) and A2 and B2 (backbone) was calculated in the unfiltered, KES and conventional temporal subtraction images for different points in time. The calculated CNR values for iodine and rib bone can be seen in Table 1 (regions A1,B1), the values between contrast agent and backbone in Table 2 (regions A2,B2). In general, the CNR for all imaging techniques fluctuates slightly over time due to the changing distribution of the contrast agent in the tissue. Still, it can be observed that KES imaging leads to the largest improvement in CNR, while conventional temporal subtraction only partially improves the CNR compared to the unfiltered images. For regions (A1,B1), KES gives a mean improvement in CNR of over 200%, while the mean CNR for conventional temporal subtraction is only about 150% better than in the unfiltered images due to strong motion artefacts. The CNR in the KES images is 21.5% higher than in the conventional temporal subtraction images. For regions (A2,B2), the increase in CNR for KES is higher, showing that for contrasted structures that lie in front of strongly absorbing structures such as the backbone, the visibility will improve substantially by using subtraction imaging. Here, on average, the CNR in the KES images is 7.7 times higher than in the unfiltered images, while the CNR in conventional temporal subtraction images is only about 6.8 times larger than in the unfiltered images. Again, the CNR in the KES images is higher than in the conventional temporal subtraction images by about 14.9%.

**Table 1:** CNR values for unfiltered, KES and conventional temporal subtraction images between regions A1 and B1 versus background region in Fig. 2. The weighted average was calculated for the four time points named in the table.

CNR(A1,B1)	16.43 s	32.80 s	49.17 s	65.54 s	weighted average
unfiltered	10.5 ± 1.8	10.8 ± 1.9	14.4 ± 2.2	13.8 ± 2.0	12.1 ± 1.0
K-edge subtraction	36.9 ± 9.4	32.0 ± 8.3	41.4 ± 9.6	37.9 ± 6.8	36.9 ± 4.1
temporal subtraction	27.4 ± 7.9	25.2 ± 9.3	36.4 ± 10.1	33.3 ± 7.9	30.4 ± 4.3

**Table 2:** CNR values for unfiltered, KES and conventional temporal subtraction images between regions A2 and B2 versus background region in Fig. 2.

CNR(A2,B2)	16.4 s	32.8 s	49.2 s	65.5 s	weighted average
unfiltered	3.7 ± 1.0	4.6 ± 1.2	6.2 ± 1.4	6.0 ± 1.4	4.9 ± 0.6
K-edge subtraction	36.4 ± 4.5	40.3 ± 6.3	42.2 ± 7.9	35.5 ± 6.2	37.8 ± 2.9
temporal subtraction	30.2 ± 6.4	32.3 ± 6.8	38.1 ± 7.6	32.3 ± 6.5	32.9 ± 3.4

## Discussion

The results suggest that K-edge subtraction imaging at a compact synchrotron source such as available at the MuCLS can provide images with improved visibility of contrasted structures in comparison to conventional non-subtraction X-ray images and with reduced artefacts compared to conventional temporal subtraction. The application KES to the conventional X-ray images improved the visibility of the contrast agent considerably. The CNR in the evaluated regions was significantly higher in the KES images compared to the unfiltered non-subtraction images and between 14.9% and 21.5% higher than in the conventional temporal subtraction images. When comparing the CNR for both regions, one can observe that it does not always improve by the same factor compared to non-subtraction images. This cannot only be explained by the emergence of artefacts in the conventional temporal subtraction images, but also with the contrast in the non-subtraction images. If the contrast between contrast agent and bone structures is already very high in non-subtraction images, the improvement in contrast in subtraction imaging does not always exceed the increase in noise and therefore the CNR may not increase^31^. This explains why the CNR for the region where the contrast agent is overlaid by the strongly absorbing backbone (A2,B2) is higher than for regions where the contrast agent is compared to rib structures which absorb the X-rays less.

Especially for clinical settings where subtraction imaging cannot be applied such as fluoroscopy of the gastrointestinal tract, KES imaging can improve the diagnostics, e.g. when looking for tiny leaks in patients with suspected intestinal perforation. However, the imaging setup was not optimized for high-speed imaging. Since the Pilatus detector has a silicon sensor with a low quantum efficiency at 33 keV, the acquisition time was limited to a minimum of 66 ms to ensure sufficient photon statistics in the images. When using a detector with a more efficient sensor material such as gadolinium oxysulfide (GadOx), gallium arsenide or cadmium telluride, the acquisition time could be further reduced to allow for even faster time scales.

Yet, the applicability of KES around the K-edge of iodine is limited due to its relatively low X-ray energy of 33.17 keV. At this energy the absorption of the X-rays in the human body is very high and thus leads to a high absorbed dose. To enable KES imaging in a clinical setting, the maximum energy of the used compact synchrotron X-ray source would have to be increased and a different contrast agent used. In the past, studies have shown that gadolinium contrast agent, which is today commonly used in MRI imaging, could also be used in X-ray imaging^33,34^, especially for patients with renal insufficiency^35,36^. The gadolinium K-edge is at 50.2 keV which would allow for dose compatible KES imaging on the human body. Currently, the source used at the MuCLS has its X-ray energy limit at 35 keV. However, several projects developing sources based on inverse Compton scattering are ongoing that will provide higher X-ray energies, such as ThomX^37^, BriXS^38^ and STAR^38^. These sources utilize higher electron energies and in case of ThomX also a larger electron storage ring than the source at the MuCLS. Higher X-ray energies would be possible at the MuCLS, too, by decreasing the laser wavelength while keeping the small footprint of the source. With these ongoing developments of inverse Compton sources, KES will become dose compatible and its application in clinically more relevant energy regime possible.

In conclusion, the results show that KES imaging at a compact synchrotron X-ray source can provide X-ray images with higher CNR compared to conventional temporal subtraction. With further development of inverse Compton sources, KES imaging has the potential for advancing existing clinical imaging techniques such as fluoroscopy where subtraction imaging currently cannot be applied and may improve diagnostics in the future.

## Methods

### KES imaging setup at the MuCLS

The experiments presented in this study were performed at the Munich Compact Light Source, which consists of a compact synchrotron X-ray source, developed and manufactured by Lyncean Technologies, USA, and two experimental end stations, built at the TUM. The Compact Light Source (CLS) provides a tunable, quasi-monochromatic X-ray beam which is produced by inverse Compton scattering of relativistic electrons and infrared laser photons^29,39^. To ensure a high X-ray flux, the electrons circulate in an electron storage ring while the laser is enhanced and stored in a high-finesse optical cavity^26^. The energy *E*_*X*_ of the produced X-ray photons in the case of backscattering and counter propagating beams is ${E}_{X}\approx 4{\gamma }^{2}{E}_{L}$, where $\gamma ={E}_{e}/(m{c}^{2})$ is the ratio of electron energy to electron rest energy and *E*_*L*_ is the laser photon energy^40^. By changing the electron energy, the X-ray energy (about 3–5% bandwidth) can be tuned in the range of 15 to 35 keV. The X-ray source’s full divergence angle is 4 mrad, resulting in a beam diameter of around 60 mm at a distance of 16.5 m from the interaction point^29^. For this experiment, the source was tuned to an X-ray spectrum with a peak at 33.7 keV with an X-ray flux of up to 3 × 10^10^ photons/s^41^.

**Figure 3:**
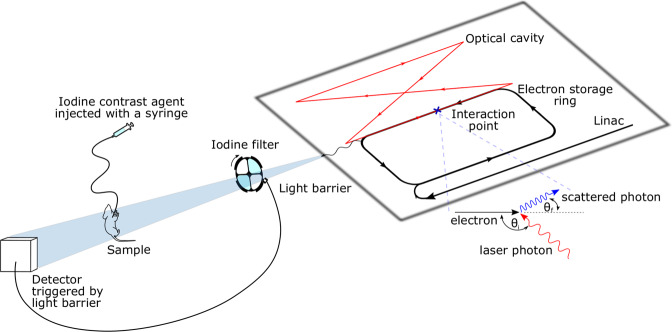
Experimental set up at the MuCLS: The quasi-monochromatic X-ray beam is produced in the compact synchrotron source by inverse Compton scattering of relativistic electrons and laser photons. Travelling to the experiment, the X-ray beam passes through the rotating filter wheel whose ports are alternatively equipped with an iodine filter and remain empty. While a port of the filter wheel is in the beam, it passes a light barrier which gates detector acquisition. The un/filtered beam then penetrates the sample and falls onto the detector. During imaging, iodine contrast agent is injected into a sample with a remotely driven syringe.

The experimental setup can be seen in Fig. 3. The quasi-monochromatic X-ray beam produced by the CLS enters the experimental set up and first hits the motorized filter wheel (custom-designed filter holder produced inhouse by additive manufacturing mounted on a FRM40, OWIS GmbH, Staufen, Germany), which has openings that are alternately empty and filled with an iodine filter. The filter wheel can rotate with up to 2600 °/s. The outer edge of the wheel is also structured to create transparent and opaque segments (corresponding to the inner openings) which pass through a fork-type light barrier (PM-L45-P-C3, Panasonic Industrial Devices SUNX Co.,Ltd., Aichi, Japan). This way, TTL trigger pulses are generated that match the time period during which the beam passes unobstructed through one of the inner openings. Inserting an iodine filter into the beam allows to switch the X-ray beam energy in the order of tens of milliseconds since the filter will almost completely absorb the part of the spectrum lying above the iodine K absorption edge. The solid iodine filter was made inhouse of an iodine-based contrast agent (Ultravist 370, Bayer Vital, Leverkusen, Germany) embedded into a polyvinylpyrrolidone (PVP) polymer matrix and constructed such that is has an effective iodine thickness of around 290 *μ*m. The filter wheel is placed in the first experimental end station at a distance of 3.5 m from the source interaction point to filter the beam when it is sufficiently small and minimize the influence of the iodine fluorescence on the images.

The sample and the detector are placed in the second experimental end station to obtain a maximum field of view. The sample, in these experiments an *ex vivo* mouse, is placed at a source-to-sample distance of 15.6 m and a sample-to-detector distance of 0.8 m. During the image acquisition, iodine-based contrast agent (Imeron 400 MCT, Bracco Imaging Deutschland GmbH, Konstanz, Germany) with an iodine concentration of 200 mg/ml is injected through a tube into the abdomen of the mouse. The syringe is fixed into a custom-built syringe holder which allowed to use a motorized linear translation to deliver a constant flow of contrast agent of 0.05 ml/s. The sample is subsequently imaged with the unfiltered and filtered X-ray beam, each image is taken with an exposure time of 66 ms. The detector, a Pilatus 200k photon counting detector (Dectris Ltd., Baden, Switzerland) with 172 × 172 *μ*m^2^ pixel size and a 1000 *μ*m thick silicon sensor, is triggered by the light barrier described earlier. It is operated in the “external enable” mode, i.e., the exposure time is as long as the external trigger pulse is active. This way, the exposure time is automatically adjusted to the optimum length for the chosen rotation speed of the filter wheel. Due to the slight cone beam, the effective pixel size in the sample plane is 164 × 164 *μ*m^2^. The mouse imaged in this experiment is euthanized in strict accordance to standard guidelines of an animal experiment proposal approved by the Institutional Animal Care and Use Committee of the Technical University of Munich. After the experiment, the organs are removed and further used according to the 3R principle (reduce, refine, replace).

### Calculation of temporal subtraction and KES images

**Figure 4:**
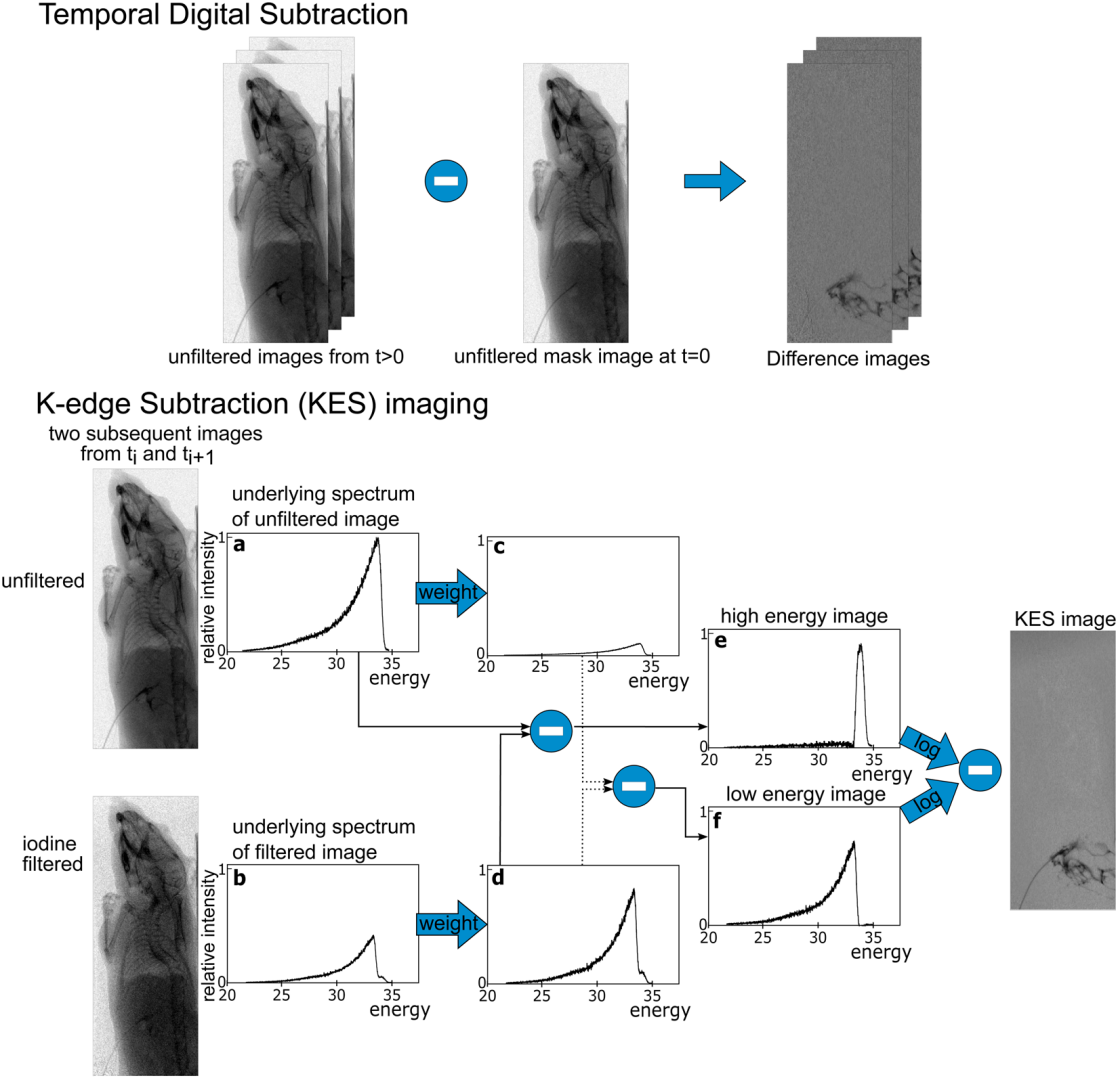
Image processing scheme for conventional temporal subtraction and K-edge subtraction (KES) imaging. First, unfiltered and iodine-filtered images are dark current, flatfield and flux corrected. In conventional temporal subtraction, the first unfiltered image is taken as a mask image and logarithmically subtracted from all following unfiltered images to generate the difference images. In KES, two subsequent unfiltered and iodine filtered images are subtracted. For these, two images with energies below and above the iodine K-edge need to be calculated. By weighting the iodine filtered image b, the loss in intensity of the part of the spectrum below the K-edge is corrected for. Subtracting this weighted iodine filtered image d from the unfiltered image a, produces a high energy image e, where only the part of the spectrum above the K-edge contributes to the image. To produce a low energy image, the remaining high energy part of the spectrum in the weighted iodine image d must be eliminated. This can be done by weighting the unfiltered image a such that it fits to the remaining high energy peak in the weighted iodine filtered image d. By subtracting the weighted unfiltered image c from the weighted iodine filtered image d, one obtains the low energy image f. The high and low energy images are then logarithmized and subtracted to obtain the KES image.

Both KES imaging and conventional temporal subtraction were performed with the images acquired. First, unfiltered and iodine-filtered images are dark current, flatfield and flux corrected. For the conventional temporal subtraction, only the unfiltered images were used, since conventional temporal subtraction uses images acquired with the same spectrum but at different points in time. Therefore, the first image acquired with the unfiltered spectrum was used as the mask image, logarithmized and then subtracted from all subsequent logarithmized images (compare Fig. 4). In contrast, in KES imaging two subsequent images with different mean energies are subtracted. However, since the used filter method does not automatically produce images with a mean energy below and above the iodine K-edge, some additional processing steps are needed as described in earlier work by Umetani *et al*.^42,43^. With the processing shown in Fig. 4, two images can be calculated which have completely separated spectra, one with a spectrum above the K-edge and the other with a spectrum below the K-edge. The first processing step is to weight the iodine filtered image Fig. 4b such that the loss in intensity of the part of the spectrum below the K-edge is corrected for. The weighting factor is determined from independent measurements of the spectra with an energy-dispersive detector (AXAS-D, Ketek GmbH, Munich, Germany). Subtracting this weighted iodine filtered image Fig. 4d from the unfiltered image Fig. 4a, produces a high energy image Fig. 4e, where only the part of the spectrum above the K-edge contributes to the image. To produce a low energy image, the remaining high energy part of the spectrum in the weighted iodine image Fig. 4d must be eliminated. This can be done by weighting the unfiltered image Fig. 4a such that it fits to the remaining high energy peak in the weighted iodine filtered image. By subtracting the weighted unfiltered image Fig. 4c from the weighted iodine filtered image Fig. 4d, one obtains the low energy image Fig. 4f. The formula for the high and low energy images are $Imag{e}_{high}=Imag{e}_{unf}-a\cdot Imag{e}_{iod}$ and $Imag{e}_{low}=a\cdot Imag{e}_{iod}-b\cdot Imag{e}_{unf}$, where $Imag{e}_{unf}$ and $Imag{e}_{iod}$ are the unfiltered and the iodine filtered images, respectively, and *a* ≈ 2.70 and *b* = 0.1. The high and low energy images are then logarithmized and subtracted to obtain the KES image. More details on the calculation of the images and noise assessment can be found in previous work^31^. Afterwards, the temporal subtraction and KES images where denoised spatially by applying a total-variation denoising algorithm using split-Bregman optimization on each image and temporally using a Gaussian filter on the temporal axis.

To simulate the movement of the sample through breathing and the movement of the organs inside the body, the images were shifted in horizontal direction. Along the time axis, these offsets followed a sinusoidal function with an amplitude of two pixels. The formula for the offsets Δ*x* between two images was Δ*x* = 2 · sin(*t*). A breathing rate of 8 breaths/min was simulated. This rate is also used for *in vivo* imaging of mice^44,45^, however only for very short periods of time (<1 min). Normally, faster breathing rates are used for *in vivo* imaging which increases the problem of artefacts in the conventional temporal subtraction even more.

### Contrast-to-noise ratio (CNR) analysis

Four regions of interest (ROIs) were selected to evaluate how the contrast-to-noise ratio (CNR) changes between the iodine contrast agent and bone structures over time. The used ROIs are highlighted in Fig. 2, where regions A1 and A2 are regions containing iodine contrast agents and B1 and B2 are the corresponding bone structures. The ROIs for iodine and bone were chosen to be 5 × 3pixels, while the larger background region had a size of 12 × 8pixels. The CNR is calculated against the noise in the background region BG according to the following formula

1 $$CNR=\frac{|{S}_{Ai}-{S}_{Bi}|}{\sqrt{{\sigma }_{back}^{2}}},$$

where *S*_*Ai*_ and *S*_*Bi*_ are the average signals in the two ROIs containing iodine contrast agent and bone structures, respectively, and *σ*_*back*_ is the standard deviation in the background region BG. For each imaging modality, the weighted average $\overline{x}$ of the CNR values over time was calculated with

2 $$\overline{x}=\frac{\mathop{\sum }\limits_{i\mathrm{=1}}^{4}{w}_{i}\cdot {x}_{i}}{\mathop{\sum }\limits_{i\mathrm{=1}}^{4}{w}_{i}}\,{\rm{with}}\,{w}_{i}=\frac{1}{\Delta {x}_{i}^{2}},$$

where *x*_*i*_ are the CNR values for each time point and Δ*x*_*i*_ are the uncertainties for each CNR value calculated by Gaussian error propagation. The uncertainty of the weighted average is:

3 $$\Delta \overline{x}=\sqrt{\frac{1}{\mathop{\sum }\limits_{i\mathrm{=1}}^{4}{w}_{i}}}\mathrm{}.$$

## Supplementary information

Supplementary Information.

Supplementary Video S1

Supplementary Video S2

Supplementary Video S3

## Data Availability

All relevant data  is publicly available from mediaTUM (https://doi.org/10.14459/2020mp1546390).
