# Supplementary material for: Dynamic K-edge Subtraction Fluoroscopy at a Compact Inverse-Compton Synchrotron X-ray Source
Source: Sci Rep. 2020 Jun 15;10:9612. doi: 10.1038/s41598-020-66414-x (PMC7295988; doi:10.1038/s41598-020-66414-x)
Supplement: Supplementary file 1 — Supplementary Information. [file 41598_2020_66414_MOESM1_ESM.pdf]

Dynamic K-edge Subtraction Fluoroscopy at a Compact Inverse-Compton Synchrotron X-ray Source

Stephanie Kulpe[(1,2)]*, Martin Dierolf[(1,2)], Benedikt Günther[(1,2)], Johannes Brantl[(1,2)], Madleen Busse[(1,2)], Klaus Achterhold[(1,2)], Bernhard Gleich[(2)], Franz Pfeiffer[(1,2,3)] and Daniela Pfeiffer[(3)]

(1) Chair of Biomedical Physics, Department of Physics, Technical University of Munich, James-Franck-Straße 1, 85748 Garching, Germany

(2) Munich School of BioEngineering, Technical University of Munich, Boltzmannstraße 11, 85748 Garching, Germany

(3) Department of Diagnostic and Interventional Radiology, Munich School of Medicine and Klinikum rechts der Isar, Technical University of Munich, Ismaninger Straße 22, 81675 München, Germany

Supplementary Information

S1-S3: Supplementary videos ‘S1_non-subtraction.mp4’, ‘S2_temporal_subtraction .mp4’ and ‘S3_KES.mp4’ show the injection of the contrast agent over time for the non-subtraction, the temporal subtraction and the K-edge subtraction images of Figure 1. The images were shifted horizontally before subtraction to simulate the movement of the mouse during breathing. After subtraction the temporal subtraction and K-edge subtraction images were shifted back to ensure that the contrast agent stays in the same position which makes the evaluation of the images easier.
